# Imputation strategies when a continuous outcome is to be dichotomized for responder analysis: a simulation study

**DOI:** 10.1186/s12874-019-0793-x

**Published:** 2019-07-23

**Authors:** Lysbeth Floden, Melanie L. Bell

**Affiliations:** Mel and Enid College of Public Health, University of Arizona, 1295 N. Martin Ave, Tucson, AZ 85724 USA

**Keywords:** Responder analysis, Clinical trials, Multiple imputation, Missing data, Missing at random

## Abstract

**Background:**

In many clinical trials continuous outcomes are dichotomized to compare proportions of patients who respond. A common and recommended approach to handling missing data in responder analysis is to impute as non-responders, despite known biases. Multiple imputation is another natural choice but when a continuous outcome is ultimately dichotomized, the specifications of the imputation model come into question. Practitioners can either impute the missing outcome before dichotomizing or dichotomize then impute. In this study we compared multiple imputation of the continuous and dichotomous forms of the outcome, and imputing responder status as non-response in responder analysis.

**Methods:**

We simulated four response profiles representing a two-arm randomized controlled trial with a continuous outcome at four time points. We omitted data using six missing at random mechanisms, and imputed missing observations three ways: 1) replacing as non-responder; 2) multiply imputing before dichotomizing; and 3) multiply imputing the dichotomized response. Imputation models included the continuous response at all timepoints, and additional auxiliary variables for some scenarios. We assessed bias, power, coverage of the 95% confidence interval, and type 1 error. Finally, we applied these methods to a longitudinal trial for patients with major depressive disorder.

**Results:**

Both forms of multiple imputation performed better than non-response imputation in terms of bias and type 1 error. When approximately 30% of responses were missing, bias was less than 7.3% for multiple imputation scenarios but when 50% of responses were missing, imputing before dichotomizing generally had lower bias compared to dichotomizing before imputing. Non-response imputation resulted in biased estimates, both underestimates and overestimates. In the example trial data, non-response imputation estimated a smaller difference in proportions than multiply imputed approaches.

**Conclusions:**

With moderate amounts of missing data, multiply imputing the continuous outcome variable prior to dichotomizing performed similar to multiply imputing the binary responder status. With higher rates of missingness, multiply imputing the continuous variable was less biased and had well-controlled coverage probabilities of the 95% confidence interval compared to imputing the dichotomous response. In general, multiple imputation using the longitudinally measured continuous outcome in the imputation model performed better than imputing missing observations as non-responders.

## Background

Clinical trials can be evaluated by differences in rates of successful response. In so-called responder analysis, subjects are classified as responders, often by dichotomizing a continuous outcome, if they improve by a specified threshold. For example, responder definitions could be a 5% change in body mass index or an improvement in symptoms by 10 points on a 100-point symptom scale. Responder analysis is commonly used with patient-reported outcomes (PROs) because results are easily interpretable to patients and other stakeholders and can lend language to drug labels and promotional claims.

When the outcome is measured for all subjects at baseline and the timepoint of interest, responder status can be calculated, and the analysis is routine. However missing data are ubiquitous in longitudinal trials and responder status cannot be determined for subjects missing the outcome. [1] One approach for handling missing data in responder analysis, recommended in the regulatory setting [2–4] is to impute subjects missing the outcome as non-responders, termed non-response imputation (NRI). However, it is a strong assumption to assume unobserved outcomes are uniformly “failures” rather than come from the distribution of subjects who do not improve. NRI can be thought of as a composite outcome of response and a dropout indicator. Methodologists warn that composite endpoints can be misleading, for example, when the components have varying degrees of severity and treatment effects of each component differ between groups. [5, 6] This could be true if dropout depended at least partly on a tolerability. For example, a cancer treatment may offer a favorable toxicity profile relative to a comparator. Using NRI, the response rate of the comparator arm more than in the treatment arm would reflect the effect of tolerability, i.e., have more non-responders, and could widening the between arm difference. While some may view NRI as a conservative approach (since the proportions of responders can only decrease), treating missing as response failure can result in unpredictable differences in proportions between treatment groups. [7, 8]

In longitudinal trials, missing observations can be intermittent, as in a missed study visit, but dropout accounts for most missing data. We focus this article on monotone missing patterns, implying that observations are observed up until one is missing and all subsequent observations are missing. Little and Rubin [9] provide a framework to describe categories of missing data mechanisms given the relationship with observed and unobserved values. When the probability of missingness is independent of the observed and unobserved data the mechanism is said to be missing completely at random (MCAR). Data are missing at random (MAR) if the probability of missingness is independent of the unobserved data after conditioning on observed data. Finally, data are considered missing not at random (MNAR) if they are neither MCAR or MAR and the missing mechanism depends on the unobserved values, given the observed data.

The MAR assumption is usually reasonable in the context of longitudinal trials and current guidance outlines a framework that includes sensitivity analyses to assess the extent to which analytic approaches are robust to missing data assumptions. [10–12] Appropriate analyses that assume MAR include mixed models using maximum likelihood estimation, extensions of generalized estimating equations (GEEs) such as weighted GEE, and multiple imputation (MI). [13, 14] Of these, MI is the only approach that can be used with any analytic model. MI is a three-stage process. First, missing values are filled *M* times by a random draw from a posterior distribution of the imputation model to generate *M* complete datasets. Secondly, the *M* datasets are analyzed via any statistical approach and thirdly, results are combined using a set of rules that accounts for the uncertainty of the imputed values. [15] The imputation model must be congenial, i.e., include the same variables, but does not have to be consistent with the substantive model. Thus, the imputation model can include variables predictive of missingness such as the outcome from intermittent timepoints, making MI a natural choice in responder analysis using a test of proportions. For these reasons we focus this paper on MI.

When a continuous outcome is ultimately dichotomized, the specifications of the imputation model come into question. Practitioners can either *impute* the missing outcome *before dichotomizing* the response (IBD) or *dichotomize* the outcome *then impute* the response (DTI). Demirtas evaluated efficiency and accuracy of the estimated proportions of responders using IBD under the multivariate normal assumption compared to DTI using a saturated binomial model for the dichotomous response indicator, and concluded that DTI was superior across most scenarios. [16] This finding is in contrast to Yoo’s work that concluded MI with GEEs performs better when the underlying continuous outcome is imputed prior to dichotomizing. [17] More generally, Von Hippel’s work supports the use of *just-another-variable* (JAV), analogous to DTI, to impute a quadratic and interaction term under a linear regression analysis model with a conceptual argument extending to the logistic setting. [18] Others demonstrated poor performance using JAV when data were MAR particularly with logistic regression [19], prompting some researchers to discourage this practice. [14]

In trial settings where the dichotomized response of a continuous outcome is of interest, there is no clear best way to handle missing data. The aim of this paper is to clarify inconsistent results in the performance of multiply imputing the IBD or DTI in responder analysis and compare with the commonly recommended non-response imputation.

## Methods

### Notation and analysis

Let the underlying continuous measure which is to be dichotomized into the response indicator be *Y*_*ij*_ for subject *i* where *i* = 1, …, *n* measured at the *j* timepoint. Measurements are repeated over time such that *j* = 1, …, *t*_*i*_ are the observed measurements for each subject and *t*_*i*_ represents the time of dropout or end of the study, *T*. Without loss of generality, assume that higher values of *Y* indicated better outcomes. Let *Y*_*ij* > 1_ − *Y*_*i*1_ = *C*_*i*_ represent change from baseline to time *j* > 1. Subject *i* is classified as a responder if *C*_*i*_ ≥ *λ* for some threshold *λ*, defined as *R*_*i*_ = *I*(*C*_*i*_ ≥ *λ*). Consider a randomized controlled trial with treatment and control arm.

The objective of responder analysis is to evaluate the difference in proportion of responders at the endpoint between treatment arms.

### Multiple imputation approach

When data have either an intermittent or monotone missing pattern, multiple imputation using the Markov chain Monte Carlo (MCMC) method and fully conditional specification (FCS, also known as imputation by chained equations) method are two popular options. [20] Both are relatively flexible to specify, straightforward to understand, and easy to apply with standard statistical software. The FCS assumes the existence of, but does not rely on, a multivariate distribution. [20] Specifically, the FCS approach assumes conditional densities for each partially observed variable and uses a corresponding regression model to sequentially generate imputations, e.g., linear regression for continuous variables and logistic regression for categorical variables. We used FCS MI for imputing both the unobserved continuous outcomes for IBD MI and the missing responder status for DTI MI, both using the continuous outcomes intermittent timepoints as auxiliary variables, and in some cases, additional covariates related to the outcome, detailed below. Thus, the comparison is not in the MI method but rather the specification of the imputation model.

In general, the FCS procedure can be described in the following steps. [21, 22] Consider a set of variables *X* = *X*_1_, …, *X*_*q*_ in the imputation model. First, starting values for unobserved measures are filled in sequentially for each variable in the order specified. Continuous variables are filled in by regressing one variable, say, *X*_1_, on the other *X*_2_, …, *X*_*q*_ covariates and using the resulting set of parameters to fill in the missing values of *X*_1_. Binary variables are filled in similarly using logistic regression. The next imputation phase replaces the filled in values with imputed values. For a set of observed values of one variable, *X*_1_, the corresponding imputation model is fit using both the observed and filled-in values of all other *q* − 1 variables as the independent variables and *X*_1_ as the dependent variable. In this study, the binary variable, *R*, is fit using logistic regression and the continuous variables, *Y*_*j*_, are fit with linear regression. The resulting set of parameters are used to impute the first set missing values. The latter two steps are repeated on the remaining *q* − 1 variables to comprise a cycle. The algorithm runs through a number of cycles updating the imputed values until convergence, at which point the current values of all *X* ’s complete the first imputed dataset. The process is repeated for *M* datasets.

To calculate the estimand *θ* using IBD MI, we imputed the missing continuous outcomes *Y*_*j*_, calculated the responder status, *R*_*i*_, estimated the difference and combined estimates using Rubin’s rules in the final step. For DTI MI, we calculated responder status prior to imputing and included the partially observed responder status, *R*_*i*_, in the imputation model. Using the imputed *R*_*i*_, we calculated the difference in proportions between treatment arms on the *M* datasets and combined using Rubin’s rules.

### Data generation

We simulated twenty-four scenarios to represent a randomized trial with two treatment arms with *N* = 200, and a continuous outcome measured at baseline and three subsequent timepoints. The scenarios described two response profiles with the same mean difference at the final assessment, six mechanisms of dropout, and two dropout rates. One response profile was linear where only treatment A was effective. In the other response profile, treatment A is effective after a period of worsening and treatment B demonstrates no effectiveness after a period of improving, hence the mean trajectories of treatment A and B cross. The third and fourth response profiles had no treatment differences at the final timepoint and were used to evaluate type 1 error.

Data for the continuous response were simulated to represent a PRO scale with equal allocation to treatment groups. Let *Y*_*ij*_ represent a continuous measure for the *i*^*th*^ individual at the *j*^*th*^ timepoint where  *j* = 1, …, 4. Specifically, data were simulated according to the underlying model:

1 $$ {Y}_{ij}=\left({\beta}_0+{b}_i\right)+{\beta}_j+{\delta}_j\ast {x}_{trt}+{\epsilon}_{ij} $$

where *x*_*trt*_ = 1 for treatment arm A and 0 for treatment arm B, *β*_*j*_ denotes the effect of the *j*^*th*^ timepoint and *δ*_*j*_ ∗ *x*_*trt*_ is the interaction of treatment group and the timepoint. Here, ${b}_i\sim N\left(0,{\sigma}_b^2\right)$ represents the random subject effect and the error term, ${\epsilon}_{ij}\sim N\ \left(0,{\sigma}_{\epsilon}^2\right)$ represents the within-subject error. The mean vectors for the linear response profile were *μ*_*A*_ = (65, 67, 69, 71)′ and *μ*_*B*_ = (65, 65, 65, 65)′ . The non-linear response profile was *μ*_*A*_ = (65, 63, 68, 71)′ and *μ*_*B*_ = (65, 67, 66, 65)′. The third and fourth response profiles to estimate type 1 error were *μ* = (65, 65, 65, 65)′ for both arms; and *μ*_*A*_ = (65, 67, 69, 71)′ and *μ*_*B*_ = (65, 63, 68, 71)′, respectively. Based on typical PRO scale data [23], we set *σ*_*b*_ = 12 and *σ*_*ϵ*_ = 7. These variance components correspond to a compound symmetric covariance structure with a within-person correlation of 0.7. Additionally, we created a normally distributed continuous correlated variable (CV) to *Y*_4_ such that ${\rho}_{CV,{Y}_4}\cong 0.3$, and the mean and standard deviation were 38.0 and 62.7 respectively.

Let *Y*_*i*4_ − *Y*_*i*1_ = *C*_*i*_ represent change from baseline to timepoint *j* = 4. To achieve 80% power to detect the difference of response rates between the two arms, the dichotomized response was defined as *R*_*i*4_ = *I*(*C*_*i*_ ≥ 12.4). Using this definition, response rates for the first and second response profiles for treatment A and B were 25.6 and 10.6, respectively. (Exploratory result using thresholds ranging from 10 to 20 produced similar trends.)

### Missing data

We used six probability models representing plausible trial scenarios to delete post-baseline observations using a MAR mechanism. Let *Z*_*ij*_ = 0 if outcome *Y*_*ij*_ is missing and 1 otherwise.

### Dropout model 1

For the first model of dropout, the probability of missing response is dependent on the value of the observed outcome at *Y*_*j* − 1_ such that $P\left({Z}_{ij}=0\right)\propto \left(1-\Phi \left({Y}_{j-1},{\hat{\theta}}_{Y_{j-1}},{\hat{\sigma}}_{Y_{j-1}}^2\right)\right)$, where *j* > 1 and Φ is the normal cumulative distribution function with mean ${\hat{\theta}}_{Y_{j-1}}$ and standard deviation ${\hat{\sigma}}_{Y_{j-1}}^2$ estimated from the data. This model represents the probability of dropout due to lack of efficacy.

### Dropout model 2

The mechanism leading to dropout can differ by treatment. [25] To model this, observations in treatment A were more likely to be missing when the outcome, *Y*_*j* − 1_, value was low such that $P\left({Z}_{ij}=0\right)\propto \left(1-\Phi \left({Y}_{j-1},{\hat{\theta}}_{Y_{j-1}},{\hat{\sigma}}_{Y_{j-1}}^2\right)\right)$, *j* > 1, and observations in treatment B were more likely to be missing when *Y*_*j* − 1_ values were high such that $P\left({Z}_{ij}=0\right)\propto \left(\Phi \left({Y}_{j-1},{\hat{\theta}}_{Y_{j-1}},{\hat{\sigma}}_{Y_{j-1}}^2\right)\right)$, *j* > 1.

### Dropout model 3

Model 3 represents missing mechanisms in the opposite direction of model 2 for the treatment arms. For example, lack of efficacy could drive dropout in a placebo arm while those on treatment may be less motivate to return to follow up when they are feeling better, i.e. improved efficacy. Here, treatment B observations were more likely to be missing when the outcome, *Y*_*j* − 1_, value was low such that $P\left({Z}_{ij}=0\right)\propto \left(1-\Phi \left({Y}_{j-1},{\hat{\theta}}_{Y_{j-1}},{\hat{\sigma}}_{Y_{j-1}}^2\right)\right)$, *j* > 1, and treatment A observations more likely to be missing when *Y*_*j* − 1_ values were high such that $P\left({Z}_{ij}=0\right)\propto \left(\Phi \left({Y}_{j-1},{\hat{\theta}}_{Y_{j-1}},{\hat{\sigma}}_{Y_{j-1}}^2\right)\right)$, *j* > 1.

### Dropout model 4

Treatment arm dropout rate can be differential. [26, 27] We modeled substantial differential dropout by including a weight term, ${w}_{x_{trt}}$, specific to treatment arm, such that $P\left({Z}_{ij}=0\right)\propto {w}_{x_{trt}}\ast \left(1-\Phi \left({Y}_{j-1},{\hat{\theta}}_{Y_{j-1}},{\hat{\sigma}}_{Y_{j-1}}^2\right)\right)$, where *w*_1_ = 0.3 and *w*_0_ = 1.

### Dropout model 5

Here, *Y*_*i*_ was set to missing with probability $P\left({Z}_{ij}=0\right)\propto \left[\frac{1}{1+{e}^{\left({b}_1{Y}_{j-1}\right)}}\right]$, where *j* > 1 and *b*_1_ = 0.01 modeling drop out due to lack of efficacy using a different mechanism than model 1.

### Dropout model 6

We simulated a repeated indicator variable representing occurrence of adverse events (AEs) to represent drug tolerability. The probability of AE depended jointly on treatment arm and occurrence of an AE at the prior visit such that for each assessment for each treatment group

$$ {p}_j^{AE}\left({x}_{trt},\gamma \right)={P}_X\left({AE}_j=1|{AE}_{j-1}=\gamma \right)\  for\ j>2 $$

where *x*_*trt*_ represents the treatment arm and *γ* represents AE status at *j* − 1. Probabilities were estimated from actual trial data and were similar to prior published event rates (Table 1). [24] For simplicity we assumed that no AEs occurred at baseline and the probability of AE at *j* = 2 was 0.3 for *x*_*trt*_ = 1 and 0.5 for *x*_*trt*_ = 0. For each subject we generated AE status at each post-baseline visit as ${AE}_{ij}\sim Bernoulli\left({p}_j^{AE}\right)$.

**Table 1:** Conditional probabilities of AEs for *j* > 2

Timepoint	*x*_*trt*_=1_,_ *γ* = 0	*x*_*trt*_=1_,_ *γ* = 1	*x*_*trt*_=0_,_ *γ* = 0	*x*_*trt*_=0_,_ *γ* = 1
*j* = 3	.2	.8	.4	.8
*j* = 4	.1	.8	.2	.8

*x*_*trt*_=1: Treatment A, *x*_*trt*_ =0: Treatment B, *γ* = 0: No AE at *j* − 1, *γ* = 1: AE at *j* − 1

The response *Y*_*i*_ was set to missing with probability $P\left({Z}_{ij}=0\right)\propto \left[\frac{1}{1+{e}^{\left({b}_1{Y}_{j-1}+{b}_2{AE}_j\right)}}\right]$, where *j* > 1 and *b*_1_ = 0.01 and *b*_2_ =  − 0.40 to model the probability of dropout due to lack of efficacy and tolerability. If *Y*_*i*_ was set to missing, all subsequent *AE* were also set to missing.

For all dropout models, we multiplied *P*(*Z*_*ij*_ = 0) by a randomly generated uniform variable and determined a cutoff value creating the overall proportion of missing responses at *j* = 4 to be 30% or 50%. If a patient was missing at any *Y*_*j* = *a*_ then all *Y*_*j* > *a*_ were set to missing.

### Analysis and comparison of methods

We determined the required number of simulated datasets per scenario, *n*_*sim*_, by estimating the standard deviation (SD) of $\hat{\theta}$ to be ≤6.0, based on exploratory simulations and setting the maximum tolerated Monte Carlo standard error (MCSE) of bias to be ≤.15. Given $MCSE(Bias)=\sqrt{\frac{Var\left(\hat{\theta}\right)}{n_{sim}}}$’ the required number of datasets was *n*_*sim*_ = 1600. [28] For each simulated dataset, we evaluated the proportions of responders in, and the difference between, each arm at *j* = 4. For IBD MI and DTI MI, all imputation models contained the group indicator, *x*_*trt*_, and the continuous outcomes *Y*_*j*_. In some imputation models, we included *CV*, a variable representing a correlated covariate to evaluate the utility of including an auxiliary variable. For DTI MI, the imputation model included the binary response variable, *R*. Scenarios using dropout model 6 also included AE status at *j* = 2, 3, 4 in the imputation model. The *M* = 30 or *M* = 50 estimates [22] of the difference in proportions and respective standard errors when 30% or 50% of responses at *j* = 4 were missing, respectively, were combined using Rubin’s Rules. [29] Sample SAS code is included in the Appendix.

We compared percent bias, coverage probability of the 95% confidence interval (CI) from multiple imputation, power, and type 1 error rate to assess the relative performance of NRI, IBD MI and DTI MI to the fully observed simulated data. We calculated percent bias of the difference as:

$$ Percent\ bias\ of\ the\ difference=\frac{\left({\overline{p}}_A-{\overline{p}}_B\right)-\left({\pi}_A-{\pi}_B\right)}{\pi_A-{\pi}_B}\ast 100 $$

where *π* represents the true proportion of responders, and $\overline{p}$ is the average proportion of responders among datasets with missing observations. Positive values represent positive biases of the estimated difference in proportions. We calculated coverage probability as the proportion of MI results where the true value was contained within the 95% CI. Power was calculated as the percentage of statistically significant group differences at the significance level of 0.05. Similarly, the type 1 error rate was calculated as the percentage of statistically significant group differences at the significance level of 0.05 when simulating a scenario with no between group difference. We assess performance of the simulation with the MCSE of bias, mean square error (MSE), standard error of the model (SE_mod_) and the empirical standard error of the difference in proportions (SE_emp_)_._ Let $\hat{\theta}={\hat{p}}_A-{\hat{p}}_B$ be the difference in proportions between groups. MSE, calculated as

$$ MSE=\frac{1}{n_{sim}}\sum \limits_{i=1}^{n_{sim}}{\left({\hat{\theta}}_i-\theta \right)}^2 $$

is a combined measure of variance and bias. SE_mod_ is the average standard error of each $\hat{\theta_i}$, and SE_emp_, is the standard error of $\hat{\theta}$, measuring the efficiency of $\hat{\theta}$_._ Simulation and analyses were conducted using SAS software version 9.4 (SAS Institute Inc., 2013).

## Results

When the response profile was linear with 30% of responses missing, bias was less than 7.3% for all MI approaches and ranged from 8.5 to − 36.7% for NRI (Table 2). Similar results were seen in the non-linear response profile (Appendix A). IBD MI had slightly lower or equal bias relative to DTI MI for all scenarios, and bias was conservative in direction, i.e., negative for 4 out of the 5 dropout models. All MI models included the continuous repeated outcomes as auxiliary variables in the imputation model. When using DTI MI, the addition of the correlated auxiliary variable reduced bias and changed the direction from positive to negative in all scenarios except when there were differential dropout rates. Including the auxiliary variable in the IBD MI model increased the negative bias in all but the scenario with differential dropout.

**Table 2:** Comparison of simulated responder analysis results using non-response imputation, impute-before-dichotomizing and dichotomize-then-impute multiple imputation^1^

Dropout model	Imputation method	% Responders Trt A	% Responders Trt B	Difference in proportions (95% CI)	% Bias	Coverage of the 95% CI	Power
1: Lack of efficacy	NRI	17.6	6.9	10.6 (1.7, 19.5)	−29.2	81.3	0.64
	DTI MI	26.5	10.7	15.9 (5.4, 26.4)	6.0	95.2	0.77
	DTI MI with CV	24.5	9.7	14.8 (4.6, 25.0)	−1.3	94.9	0.74
	IBD MI	25.7	10.8	14.9 (4.5, 25.3)	−0.6	95.2	0.70
	IBD MI with CV	24.1	9.9	14.1 (4.0, 24.3)	−5.7	94.3	0.69
2: Differing mechanism	NRI	17.6	7.9	9.6 (0.3, 18.7)	− 35.7	77.2	0.55
	DTI MI	26.5	10.5	16.0 (5.5, 26.5)	6.7	94.8	0.77
	DTI MI with CV	24.7	9.8	14.8 (4.7, 25.0)	−1	94.3	0.74
	IBD MI	25.7	10.8	14.9 (4.5, 25.3)	−0.7	94.9	0.69
	IBD MI with CV	24.2	10.1	14.1 (3.9, 24.3)	−5.8	94.5	0.68
3: Differing mechanism, reversed	NRI	18.3	6.9	11.4 (2.4, 20.4)	−24.1	86.3	0.69
	DTI MI	26.1	10.5	15.5 (5.1, 26.0)	3.7	93.4	0.74
	DTI MI with CV	24.2	9.7	14.5 (4.4, 24.6)	−3.4	93.4	0.72
	IBD MI	25.8	10.8	15.0 (4.6, 25.5)	0.2	94.1	0.70
	IBD MI with CV	24.2	10.0	14.2 (4.0, 24.4)	−5.4	93.4	0.68
4: Differential dropout rates	NRI	21.5	5.3	16.2 (7.2, 25.3)	8.5	93.8	0.94
	DTI MI	26.0	10.8	15.2 (4.8, 25.7)	1.8	93.8	0.71
	DTI MI with CV	24.8	9.1	15.6 (5.6, 25.7)	4.5	93.3	0.79
	IBD MI	25.6	10.9	14.7 (4.3, 25.1)	−1.8	94.5	0.69
	IBD MI with CV	24.6	9.4	15.2 (5.1, 25.3)	1.7	94.8	0.77
5: Lack of efficacy, sensitivity of mechanism	NRI	16.5	6.7	9.7 (1.1, 18.4)	−35	75.4	0.59
	DTI MI	26.6	10.5	16.1 (5.6, 26.5)	7.1	93.7	0.76
	DTI MI with CV	24.4	9.6	14.8 (4.6, 24.9)	−1.5	94.3	0.72
	IBD MI	25.8	10.8	15.0 (4.6, 25.5)	0.4	94.3	0.68
	IBD MI with CV	24.0	9.9	14.2 (4.0, 24.3)	−5.5	93.6	0.67
6: Lack of efficacy and tolerability	NRI	18.0	7.1	10.9 (2.0, 19.9)	−27.1	83.8	0.67
	DTI MI	26.3	10.6	15.7 (5.2, 26.2)	4.7	93.7	0.77
	DTI MI with CV	24.4	9.7	14.7 (4.6, 24.9)	−1.9	93.8	0.75
	DTI MI with *AE*	26.5	11.0	15.5 (5.0, 26.0)	3.4	93.3	0.74
	IBD MI	25.6	10.8	14.8 (4.4, 25.2)	−1.2	93.0	0.69
	IBD MI with CV	24.1	10.0	14.2 (4.0, 24.3)	−5.5	93.8	0.69
	IBD MI with *AE*	25.7	10.9	14.8 (4.3, 25.2)	−1.5	93.2	0.69

NRI: Non-response imputation; DTI MI: Dichotomize then impute multiple imputation; IBD MI: Impute before dichotomizing multiple imputation; CV: Correlated variable; AE: Adverse event status

^1^ Results are from a linear response profile with 30% data missing at random, *N* = 200. In fully observed data, % responders in Treatment A and B was 25.6 and 10.6, respectively for a difference of 15.0 and power = 0.80

The probability of dropout in model 6 was related to both treatment arms, through AE status, and outcome score. Including AE status at *j* = 2, 3, 4 in the imputation model negligibly reduced bias with DTI MI, and maintained a similar level of bias with IBD MI, compared to no auxiliary variables.

NRI suffered from high negative bias and substantial loss of power to detect differences in all but one scenario. The proportion of responders per treatment arm were always underestimated because missing observations were classified as non-responders. When the dropout mechanism affected the two arms differentially (model 4), NRI produced a positively biased difference estimate.

When 50% of responses were missing with the linear response profile, IBD MI had less bias relative to DTI MI without the use of *CV* for all scenarios, and bias was negative in direction for 5 of the 6 dropout models (Table 3). Specifically, bias with DTI MI (with no auxiliary variables) ranged from − 21.8 to 11.0. Under the same conditions, the bias of IBD MI ranged from − 6.9 to 0.7. In general, power to detect treatment differences was lower using IBD MI compared to DTI MI.

**Table 3:** Comparison of simulated responder analysis results when 50% responses are missing using non-response imputation, impute-before-dichotomizing and dichotomize-then-impute multiple imputation^1^

Dropout model	Imputation method	% Responders Trt A	% Responders Trt B	Difference in proportions (95% CI)	% Bias	Coverage of the 95% CI	Power
1: Lack of efficacy	NRI	12.8	4.8	8.0 (0.3, 15.7)	− 46.8	55.6	0.52
	DTI MI	27.5	11.2	16.3 (5.7, 26.9)	8.8	91.5	0.72
	DTI MI with CV	24.2	9.7	14.5 (4.4, 24.6)	−3.2	90.6	0.66
	IBD MI	25.8	11.1	14.8 (4.3, 25.2)	−1.5	94.1	0.59
	IBD MI with CV	23.3	9.7	13.6 (3.5, 23.6)	−9.6	92.9	0.56
2: Differing mechanism	NRI	12.8	6.3	6.6 (−1.4, 14.6)	− 56.2	45.5	0.37
	DTI MI	27.5	10.9	16.6 (6.1, 27.2)	11.0	86.9	0.71
	DTI MI with CV	24.6	9.8	14.8 (4.7, 25.0)	− 1.2	88.7	0.65
	IBD MI	25.9	11.1	14.8 (4.4, 25.3)	−1.1	92.9	0.58
	IBD MI with CV	23.6	9.9	13.7 (3.6, 23.8)	−8.6	92.2	0.56
3: Differing mechanism, reversed	NRI	13.9	4.8	9.0 (1.1, 16.9)	− 39.8	66.5	0.62
	DTI MI	26.7	11.0	15.7 (5.1, 26.2)	4.5	85.4	0.64
	DTI MI with CV	23.9	9.7	14.2 (4.1, 24.2)	−5.4	86.1	0.61
	IBD MI	26.1	11.1	15.1 (4.6, 25.6)	0.7	92.0	0.58
	IBD MI with CV	23.6	9.8	13.8 (3.7, 23.8)	−8.1	91.1	0.55
4: Differential dropout rates	NRI	18.3	1.8	16.5 (8.5, 24.4)	10.0	93.9	0.99
	DTI MI	26.2	14.5	11.7 (0.9, 22.5)	−21.8	77.5	0.48
	DTI MI with CV	24.0	11.1	12.9 (2.7, 23.1)	−13.8	84.1	0.58
	IBD MI	25.7	11.8	13.9 (3.4, 24.5)	−6.9	92.8	0.49
	IBD MI with CV	23.8	9.4	14.4 (4.4, 24.4)	−3.7	94.4	0.60
5: Lack of efficacy, sensitivity of mechanism	NRI	13.7	5.6	8.1 (0.1, 16.1)	− 45.9	59.9	0.53
	DTI MI	26.9	10.7	16.2 (5.7, 26.7)	8.1	91.9	0.72
	DTI MI with CV	24.2	9.5	14.7 (4.6, 24.8)	−2.1	92.6	0.67
	IBD MI	25.9	10.9	15.0 (4.5, 25.4)	−0.2	94.1	0.62
	IBD MI with CV	23.5	9.8	13.8 (3.7, 23.8)	−8.1	93.6	0.60
6: Lack of efficacy and tolerability	NRI	13.2	4.9	8.3 (0.5, 16.1)	− 44.6	59.5	0.57
	DTI MI	26.9	11.0	15.9 (5.4, 26.5)	6.1	91.3	0.68
	DTI MI with CV	24.1	9.7	14.4 (4.3, 24.5)	−4.0	91.2	0.65
	DTI MI with *AE*	27.4	11.9	15.5 (4.8, 26.2)	3.4	90.1	0.63
	IBD MI	25.8	11.1	14.7 (4.2, 25.2)	−2.0	93.3	0.59
	IBD MI with CV	23.5	9.8	13.6 (3.6, 23.7)	−9.0	92.5	0.57
	IBD MI with *AE*	26.0	11.4	14.6 (4.1, 25.1)	−2.7	93.6	0.57

NRI: Non-response imputation; DTI MI: Dichotomize then impute multiple imputation; IBD MI: Impute before dichotomizing multiple imputation

^1^Results are from a linear response profile with 50% data missing at random, N = 200. In fully observed data, % responders in Treatment A and B was 25.6 and 10.6, respectively for a difference of 15.0 and power = 0.80

Coverage probabilities of 95% confidence for all MI approaches ranged from 93.2 to 95.3% when 30% of the responses were missing (Table 2). When 50% of responses were missing, the coverage probabilities when imputing the continuous response were closer to the nominal level of 95% compared to imputing the dichotomized response, ranging from 90.1 to 94.4% and 77.5 to 92.6%, respectively (Table 3). NRI coverage was lower than the MI approaches in all scenarios except for when there was differential dropout. Although IBD MI generally had lower power to detect treatment differences compared to DTI MI, the difference was negligible. NRI was more precise as measured through the SE_emp_ of the difference in proportions between groups, compared to all MI approaches (Table 4). However, as a function of the high levels of bias, NRI performed poorly in terms of MSE compared to the MI approaches. The MCSE of bias was between 0.12–0.14, less than our tolerated level of uncertainty, when 30% of responses were missing. NRI had higher precision estimating the group difference, compared to the other approaches as seen with the lower SE_emp_. The SE_mod_ was similar to the SE_emp_ suggesting bias of SE_emp_ is not a concern.

**Table 4:** Comparison of Monte Carlo standard error, mean squared error, model and empirical standard error using non-response imputation, impute-before-dichotomizing and dichotomize-then-impute multiple imputation^1^

Dropout model	Imputation method	MCSE	MSE	SE_mod_	SE_emp_
1: Lack of efficacy	NRI	0.12	40.84	4.46	4.65
	DTI MI	0.13	28.94	5.99	5.31
	DTI MI with CV	0.13	27.31	5.75	5.23
	IBD MI	0.14	29.26	6.14	5.41
	IBD MI with CV	0.13	28.27	5.84	5.25
2: Differing mechanism	NRI	0.12	50.99	4.66	4.72
	DTI MI	0.14	31.11	6.10	5.49
	DTI MI with CV	0.13	29.10	5.84	5.39
	IBD MI	0.14	30.03	6.14	5.48
	IBD MI with CV	0.13	29.70	5.93	5.38
3: Differing mechanism, reversed	NRI	0.12	35.34	4.60	4.72
	DTI MI	0.14	31.63	6.03	5.60
	DTI MI with CV	0.14	30.36	5.72	5.49
	IBD MI	0.14	31.39	6.17	5.60
	IBD MI with CV	0.14	30.30	5.95	5.45
4: Differential dropout rates	NRI	0.12	24.27	4.67	4.76
	DTI MI	0.14	31.26	6.00	5.59
	DTI MI with CV	0.13	29.13	5.69	5.36
	IBD MI	0.13	28.54	6.10	5.34
	IBD MI with CV	0.13	27.13	5.79	5.20
5: Lack of efficacy, sensitivity of mechanism	NRI	0.12	48.88	4.41	4.63
	DTI MI	0.14	31.97	6.13	5.56
	DTI MI with CV	0.14	29.59	5.89	5.44
	IBD MI	0.14	31.04	6.23	5.57
	IBD MI with CV	0.14	30.00	6.01	5.42
6: Lack of efficacy and tolerability	NRI	0.12	39.14	4.58	4.75
	DTI MI	0.14	30.73	6.00	5.50
	DTI MI with CV	0.13	28.74	5.73	5.36
	DTI MI with *AE*	0.14	32.73	5.84	5.70
	IBD MI	0.14	31.44	5.94	5.61
	IBD MI with CV	0.14	30.13	5.73	5.43
	IBD MI with *AE*	0.14	31.43	5.91	5.60

MCSE: Monte Carlo standard error; MSE: Mean squared error; SE_mod_: Average standard error of the risk difference; SE_emp_: Empirical standard error of the risk difference; NRI: Non-response imputation; DTI MI: Dichotomize then impute multiple imputation; IBD MI: Impute before dichotomizing multiple imputation; CV: Correlated variable; AE: Adverse event status

^1^ Results are from a linear response profile with 30% data missing at random, N = 200. In fully observed data, % responders in Treatment A and B was 25.6 and 10.6, respectively for a difference of 15.0 and power = 0.80

Type 1 error rate was controlled at less than 5% for both multiple imputation strategies, reported in Table 5. When dropout rates differed between groups (model 4), NRI had type 1 error rates ranging from 0.16 to 0.31, suggesting false positives are of concern.

**Table 5:** Type 1 error rate for non-response imputation, dichotomizing before multiply imputing, and multiply imputing before dichotomizing when missing =30%^1^

	Null response profile 1		Null response profile 2	
	Dropout model 1	Dropout model 4	Dropout model 1	Dropout model 4
NRI	0.06	0.16	0.05	0.31
DTI MI	0.03	0.04	0.03	0.04
DTI MI with CV	0.03	0.04	0.03	0.04
IBD MI	0.02	0.02	0.03	0.03
IBD MI with CV	0.03	0.02	0.03	0.04

NRI: Non-response imputation; DTI MI: Dichotomize then impute multiple imputation; IBD MI: Impute before dichotomizing multiple imputation

^1^Using Dropout model 1 and 4

^2^ Null response profile 1: =(65, 65, 65, 65)′; Null response profile 2: *μ*_*A*_ = (65, 67, 69, 71)′ and *μ*_*B*_ = (65, 63, 68, 71)′

The non-linear response profile demonstrated very similar results overall, as shown in the Appendix.

### Application to a clinical trial

We applied the above imputation approaches to data adapted from a Phase III randomized, double-blind clinical trial in patients with major depressive disorder. The trial evaluated efficacy of duloxetine 40 mg/d and 80 mg/d versus placebo and a comparator, paroxetine 20 mg/d, to treat emotional and physical symptoms in depressed patients. [30] Details of the original trial design are reported in Goldstein et al. [30] For the purpose of this study, we considered a publicly available dataset modified from the original trial data. [31] The trial included four parallel arms; the modified dataset has two arms: the original placebo arm and a “treatment” arm consisting of a random sample of patients from the three active drug arms. At 6 weeks post randomization, 75% of the patients remained in the study. To further illustrate the effect of imputation choice, we used a MAR mechanism (Dropout model 1) to identify observations to omit so that 60% of patients have outcome values at week 6. The outcome was the total score on the 17-item Hamilton depression rating scale (HAMD-17), measured at baseline and weeks 1, 2, 4, and 6 after randomization. Lower scores indicate less severity; negative change scores indicate improvement. We conducted a responder analysis using a meaningful change threshold of 6 points to assess the proportions of patients who improved at 6 weeks post-baseline, as this threshold coincides with common categories of depression severity, e.g., the difference between mild and moderate depression is 6 points.

### Case study results

At baseline *N* = 172 subjects (*n* = 84 in the treatment group and *n* = 88 in the control group) had complete HAMD-17 total scores. The difference in proportions of responders at week 6 was 19.1% (*p* = 0.009), 21.9% (p = 0.009) and 21.1% (*p* = 0.007) estimated using NRI, IBD MI and DTI MI, respectively (Table 6). When the number of patient dropouts was increased to 40%, the difference in proportions was reduced from 19.1 to 13.1% (*p* = 0.064), remained similar at 21.9 and 22.6% (*p* = 0.007), or increased from 21.1 to 24.6% (*p* = 0.002) when using NRI, IBD MI and DTI MI, respectively, compared to the original data. We repeated the random sampling using dropout model 1 three times and saw similar results. These results show that as missingness increased, IBD estimates remained similar. NRI estimates decreased (and were no longer able to detect statistically significant differences) and DTI MI estimates increased slightly. Using the IBD method, 56.3% of patients in the treatment arm improved at least as much as 6 points in the HAMD-17 depression scale compared to 36.3% of those in the placebo arm for a between group difference of responders of 21.9 (CI: [5.3, 36.6], *p* = 0.009).

**Table 6:** Comparison of imputation results for a clinical trial example. Treatment arm: *n* = 84; Placebo arm: *n* = 88

% Missing		NR imputation responders*		IBD imputation responders*		DTI imputation responders*	
	Arm	%	Difference	%	Difference	% (95% CI)	Difference
25%	Drug	46.4	19.1 (*p* = 0.009)	56.3 (45.9, 68.7)	21.9 (5.3, 36.6) (*p* = 0.009)	56.6 (45.7, 67.5)	21.1 (5.8, 36.5) (*p* = 0.007)
	Placebo	27.3		36.3 (25.7, 47.0)		35.5 (24.6, 46.3)	
40%	Drug	38.1	13.1 (*p* = 0.064)	60.5 (48.2, 72.8)	22.6 (6.2, 39.1) (*p* = 0.007)	59.2 (48.1, 70.3)	24.6 (8.9, 40.5) (*p* = 0.002)
	Placebo	25.0		37.8 (26.2, 49.5)		34.6 (23.5, 45.7)	

*Response is defined as improvement ≥6 on the HAMD-17 total score from baseline to week 6

## Discussion

When continuous data are collected in longitudinal trials with the ultimate interest in differences of a binary response, imputing missing as non-response produces positively and negatively biased estimates. Multiply imputing before dichotomization is often slightly less biased than dichotomizing then imputing but both methods perform well when 30% of the responses are missing. When there are higher rates of missing outcomes, dichotomizing before imputing produced estimates with over 10% bias in three scenarios. When applied to real trial data where the true difference in proportions is unknown, the method of imputing prior to dichotomizing produced similar estimates when both 25 and 40% of observations at the endpoint were missing.

Literature addressing IBD and DTI has been contradictory. One reason could be the choice in MI method. For example, Demirtas used a saturated multinomial model to impute the binary outcome. [16] While statistically sound, this MI approach is not readily available in standard statistical software. Another study using the Markov chain Monte Carlo (MCMC) method comparing IBD MI and DTI MI prior to assessing binary outcomes longitudinally via GEEs found an advantage to imputing before dichotomizing, consistent with the work of Yoo. [17] One distinguishing feature of our study was the use of the continuous *Y*_*j*_’s as auxiliary variables in the imputation model making the MAR assumption more likely if they are predictive of missingness, the outcome, or both. [14, 25]

The use of auxiliary variables in addition to the outcomes from interim timepoints in the imputation models provided limited usefulness. It is likely that the correlation between *CV* and the outcome was not strong enough to systematically increase precision. Further, adverse events were not related to the outcome after conditioning on the treatment group. The use of auxiliary variables are generally useful to reduce the standard error when highly correlated with the outcome or reduce bias when correlated with the outcome and missingness. [22]

It is unclear why NRI is a recommended strategy in light of the highly biased estimates produced in this simulation and others. [7, 8, 32, 33] Practitioners may erroneously believe that NRI always produces conservative results. Indeed, the NRI can only underestimate proportions of responders in treatment groups. However, when the difference in proportions is of interest, which is usually the case, using NRI when there is differential dropout can yield erratic results including positively biased estimates as shown in model 4. [7, 26] Further warnings include those related to composite endpoints [5, 6] and single imputation methods which underestimate the uncertainty of the missing data in the form of overly precise standard errors. [13, 34]

This study aimed to determine the optimal approach to imputing missing observations for responder analysis when a continuous variable is dichotomized. However, it is impossible to simulate all scenarios that could occur in real settings. We simulated outcomes under a normal distribution which may not always happen. For example, the baseline measure will not be normally distributed if the measure is also an inclusion criterion and subjects must meet a cutoff value. Many outcomes, such as PROs, are measured ordinally and imputing a continuous version via a linear regression could produce values not possible on the original scale. Data here were simulated to be MAR yet in real settings missing may be MNAR or a mixture of mechanisms.

## Conclusion

We compared imputation methods for missing outcomes in a responder analysis. MI approaches using the longitudinally measured continuous outcome as auxiliary variables performed better than imputing missing observations as failures. Differences in proportions of responders between arms, bias, coverage probabilities of the 95% confidence interval, and other performance measures were similar for both MI approaches with moderate rates of missingness. With high rates of missingness, imputing the continuous outcome prior to dichotomizing was less biased and provided better coverage probability than imputing the already transformed response. Trialists conducting responder analysis by dichotomizing a continuous outcome can benefit from these findings.

## Data Availability

The dataset analyzed as the case study during the current study is available at http://www.missingdata.org.uk. The simulated datasets analysed during the current study are available from the corresponding author on reasonable request.

## Abbreviations

AE: Adverse events
CI: Confidence interval
CV: Correlated variable
DTI: Dichotomize then impute
FCS: Full conditional specification
GEE: Generalized estimating equation
HAMD: Hamilton Depression Rating Scale
IBD: Impute before dichotomizing
JAV: Just another variable
MAR: Missing at random
MCAR: Missing completely at random
MCMC: Markov chain Monte Carlo
MI: Multiple imputation
MNAR: Missing not at random
MSE: Mean squared error
NRI: Non-response imputation
PRO: Patient reported outcome
SD: Standard deviation
SE_emp_: Standard error, empirical
SE_mod_: Standard error, model
